# Seroprevalence and Risk Factor Assessment of Foot and Mouth Disease Virus in the Pakistan–Afghanistan Border Region

**DOI:** 10.3390/pathogens15040407

**Published:** 2026-04-08

**Authors:** Abdul Kabir, Asghar Ali Kamboh, Muhammad Abubakar, Kinkpe Lionel, Abdulkareem Mohammed Matar

**Affiliations:** 1 Department of Veterinary Microbiology, Faculty of Animal Husbandry and Veterinary Sciences, Sindh Agriculture University, Tandojam 70060, Pakistan; 2 National Veterinary Laboratory (NVL), Ministry of National Food Security & Research, Islamabad 45500, Pakistan; mabnvl@gmail.com; 3 College of Animal Science and Technology, Northwest Agriculture and Forestry University, Taicheng Road, Yangling, Xianyang 712100, China; 4 Department of Animal Production, College of Food and Agriculture Sciences, King Saud University, P.O. Box 2460, Riyadh 11451, Saudi Arabia

**Keywords:** border area, ELISA, FMD, large ruminants, prevalence, serotypes

## Abstract

Foot and mouth disease (FMD) is a highly contagious transboundary viral disease affecting livestock, causing significant economic losses. This sero-epidemiological study investigated FMD distribution and associated risk factors in cattle and buffaloes along the Pakistan–Afghanistan border. A total of 800 serum samples were collected from cattle (*n* = 610) and buffaloes (*n* = 190) and tested for antibodies against FMD viral structural proteins (SP) and non-structural proteins (NSP) using ELISA. Overall, 35.25% (282/800) of samples were NSP-positive, indicating natural infection. Serotype-specific analysis showed serotype O as the most prevalent (66.1%), followed by serotype A (50%) and Asia-1 (32%). Cattle exhibited higher FMD prevalence (37%; 95% CI: 33–40) than buffaloes (30%; 95% CI: 23–37). Significant spatial variations in SP and NSP Seroprevalence were observed across different areas. Risk factor analysis identified male sex, young age (1–2 years), crossbred and exotic breeds, summer season, large herd size, smallholders subsistence production systems, poor body condition, and animal movement as factors associated with significantly higher (*p* < 0.05) FMD circulation. These findings indicate that FMD is highly endemic in the border region and highlight the critical need for government-led mass vaccination campaigns, targeted risk-based surveillance, and stringent movement control to mitigate disease spread. Implementation of such control strategies is essential to safeguard livestock health and protect the regional economy from substantial losses.

## 1. Introduction

Foot and mouth disease (FMD) is a highly contagious disease of ungulates caused by the foot and mouth disease virus (FMDV). A member of the *Picornaviridae* family [1], FMDV exists as seven distinct serotypes: O, A, Asia-1, C, and South African Territories (SAT) 1, 2, and 3 [2]. The severity of FMDV infection varies depending on factors such as the animal’s health, immune status, and level of virus titer in the blood [2,3]. The disease causes severe economic losses to livestock production and trade due to reduced animal productivity, increased mortality, and restricted market access [4]. Despite global efforts to control FMD through vaccination and surveillance, the disease remains endemic in many regions, particularly in Asia and Africa [5].

Pakistan is one of the countries where FMD is endemic and poses a serious threat to the livestock sector, which contributes about 11% to the national Gross Domestic Product (GDP) [4]. According to the Food and Agriculture Organization (FAO), Pakistan lacks proper vaccination strategies, vaccine supply chains, and quality control mechanisms for FMD control [4,6]. Moreover, Pakistan shares borders with several countries where FMD is also prevalent, such as Afghanistan, India, China, Iran, and Tajikistan. The frequent cross-border movement of animals facilitates the transmission and spread of FMDV among different populations [2,7].

The risk factors of FMD seropositivity can be classified into intrinsic and extrinsic factors. Intrinsic factors are related to the characteristics of the individual animals, such as age, sex, health condition, and breed. Extrinsic factors are related to the environmental and management factors that influence the exposure and transmission of FMDV, such as farming system, herd size and composition, animal movement, wildlife contact, outbreak location, farmer awareness, agroecological status, and communal grazing and watering practices [7].

Significant livestock movement occurs throughout the year due to trade and market demands in Pakistan. A notable contributor to this movement is the annual religious festival of Eid al-Adha, during which large numbers of animals are transported across provinces and borders without proper quarantine or vaccination, creating high-risk conditions for FMDV spread [8]. Although several studies have investigated the serodiagnosis and risk factors of FMD in ruminants in Pakistan [9] and neighboring countries [2] there is still insufficient data on large ruminants in the Pakistan–Afghanistan border regions where animal movement is high. A study by Kabir et al. [10] reported 67% seroprevalence of FMD in the northern border region of Pakistan; however, the study did not explore the serotypes circulated in the area [11].

Therefore, this study aimed to address this knowledge gap by employing structural proteins (SP) and (NSP) ELISA techniques to estimate seroprevalence and identify risk factors of FMD in large ruminants within the Pakistan–Afghanistan border region. The research aims to offer valuable insights into the current FMD status in the area and propose opportunities for enhancing FMD control by implementing best practices and international standards. The findings have the potential to contribute to the development of a comprehensive national strategy for FMD control in Pakistan, ultimately benefiting the livestock industry and livelihoods of millions of people.

## 2. Materials and Methods

### 2.1. Ethical Statement

All animal experimental procedure adhered to international ethical standards and were conducted in accordance with the guidelines of the Institutional Animal Care and Use Committee (IACUC). Formal approval was received from the Directorate of Advanced Studies, Sindh Agriculture University (SAU), Tandojam (Approval No. DAS/1425 of 2021). Written informed consent was obtained from all animal owners prior to sample collection.

### 2.2. Study Area

**Figure 1:**
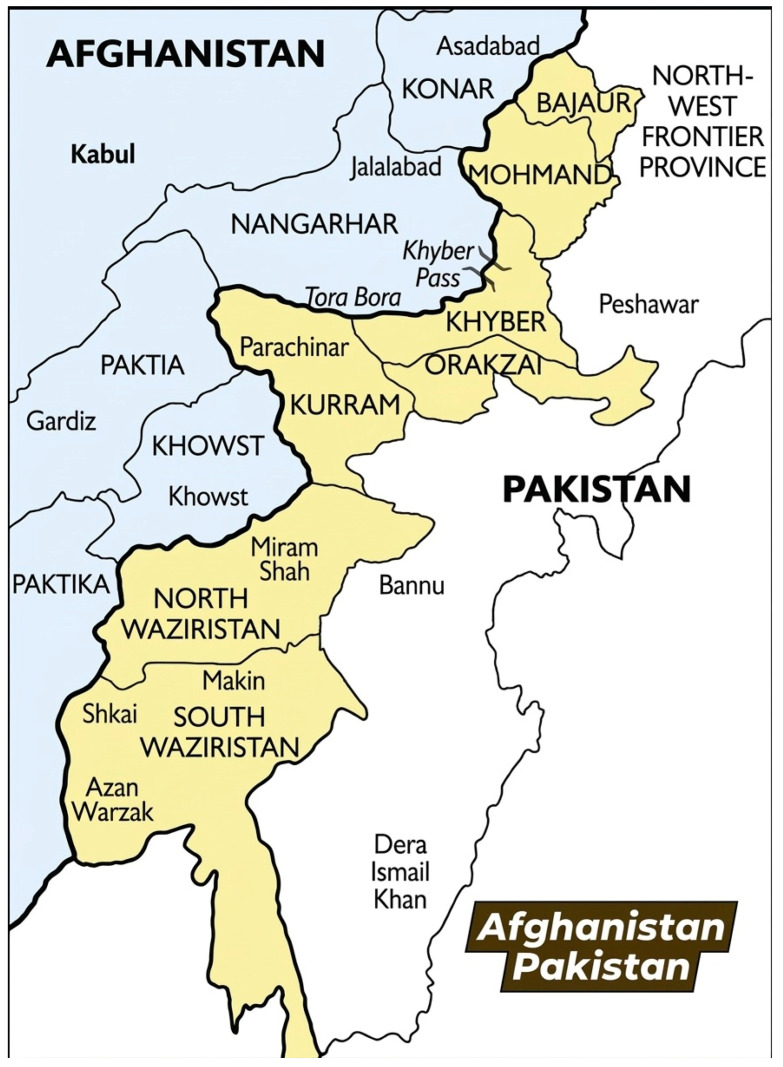
Map illustrating the study area along the Pakistan–Afghanistan border. The highlighted districts, including Bajaur, Mohmand, Khyber, Kurram, North Waziristan, and South Waziristan, were selected for sample collection and epidemiological investigation.

This study investigated the occurrence of foot and mouth disease (FMD) among cattle and buffaloes in five regions along the Pakistan–Afghanistan border: Khyber, Bajour, Mohmand, Kuram, and South Waziristan. These regions cover an area of approximately 27,220 km^2^ (Figure 1) and have a cattle/buffalo population of 1.37 million, according to the 2021–2022 official animal census [12]. Local administration and veterinarians report frequent cross-border movement of animals, which makes these regions particularly vulnerable to various transboundary diseases.

### 2.3. Sample Size

A total of 800 samples from large ruminants were collected across the study area: Khyber (*n* = 165), Bajour (*n* = 164), Mohmand (*n* = 164), Kuram (*n* = 147), and South Waziristan (*n* = 160). The sample size was calculated using the formula described by Thrusfield [13] with the following parameters: expected prevalence of FMDV at 50% (to maximize sample size), desired precision of 5%, and confidence level of 95%. The minimum required sample size was calculated as 384. To account for potential non-response and improve precision across multiple districts, the total sample size was increased to 800. This extensive sampling aimed to capture the FMD prevalence and its impact on livestock in these high-risk regions.

### 2.4. Animal Selection Criteria

The study involved cattle and buffaloes from the border region of Pakistan and Afghanistan. The cattle included indigenous (i.e., Achai, Sahiwal, Red Sindhi, Dhanni, and Cholistani), exotic (viz., Holstein-Friesian) and crossbred types; while the buffaloes comprised indigenous (i.e., Azakheli, Kundi, and Nili-Ravi) and crossbred breeds. The sample size was estimated using the formula of Thrusfield [13]. A multistage cluster sampling design was initially adopted, with tehsils selected as primary sampling units and villages as clusters. However, due to security constraints and difficult terrain in the Pakistan–Afghanistan border region, accessible villages within each cluster were selected based on practical feasibility and farmer willingness to participate. This hybrid approach allowed for broad geographic coverage while acknowledging the practical limitations of sampling in a high-risk area. A total of 29 tehsils were included in the sample collection.

### 2.5. Blood Collection

In this cross-sectional study, a convenient sampling procedure was used to collect samples from a total of 800 animals, comprising 610 cattle and 190 buffaloes, from the Pakistan–Afghanistan border region. Blood samples, approximately 5 mL, were collected from the jugular vein of each animal using sterile syringes and needles. The collected blood was immediately transferred to gel-barrier tubes, each labeled with a unique ID code for identification. These tubes were then centrifuged to separate the serum. The serum was carefully collected and placed into 2 mL Eppendorf tubes, which were labeled and subsequently frozen.

To maintain the integrity of the samples, they were kept frozen at −20 °C until they were transported to the laboratory. During transportation, the samples were kept cold to prevent degradation. Upon arrival at the National Veterinary Laboratory (NVL) in Islamabad, Pakistan, the samples were stored at −20 °C until further analysis could be conducted.

### 2.6. ELISA

In this study, two complementary ELISA formats were employed. The SP-ELISA (IZSLER, Brescia, Italy) detects serotype-specific antibodies against structural proteins, indicating either vaccination or past infection. The NSP-ELISA (IZSLER, Brescia, Italy) detects antibodies against non-structural proteins, allowing identification of natural FMDV infection regardless of vaccination status. Both kits have been validated with reported sensitivity and specificity exceeding 95% for their respective targets [14]. Samples were considered positive if the optical density (OD) value was ≥50% of the positive control, in accordance with the manufacturer’s instructions (IZSLER, Brescia, Italy). Positive and negative controls were run in duplicate on each plate; results were accepted only if control values fell within the manufacturer’s specified ranges. Samples with OD values within ±10% of the cut-off threshold were re-tested in duplicate; if the repeat result remained within this range, the sample was classified as negative.

At the time of sampling, there was no coordinated mass vaccination campaign in the study area. Vaccination was practiced sporadically by individual farmers, primarily in market-oriented herds. Therefore, SP-ELISA positivity reflects a combination of natural infection and vaccination, while NSP-ELISA positivity specifically indicates natural infection. The NSP-ELISA (IZSLER, Brescia, Italy) is a validated DIVA (Differentiating Infected from Vaccinated Animals) assay, as it detects antibodies against non-structural proteins that are not present in inactivated vaccines [14]

### 2.7. Data Analysis

Following data collection, the results were meticulously recorded and organized in a Microsoft Excel Spreadsheet for systematic analysis. To derive meaningful insights, analytical statistics were computed using Minitab 16 software (Minitab Inc., State College, PA, USA) and SPSS version 26.0 (IBM Corp., Armonk, NY, USA) Univariable and multivariable logistic regression analyses were performed to identify independent risk factors associated with FMDV seropositivity. Variables with *p* < 0.20 in univariable analysis were included in the multivariable model. Results are presented as adjusted odds ratios (aOR) with 95% confidence intervals (CI). Apparent seroprevalence in sampled animals was estimated by calculating the 95% CI using the Wilson score method for binomial proportions [15]. The results were considered significant when probability was <0.05. These statistical analyses helped to reveal valuable patterns and trends in the dataset, enhancing our understanding of the disease’s epidemiology in the studied population [16,17].

## 3. Results

### 3.1. Overall Seroprevalence of FMDV

SP-ELISA detects antibodies against structural proteins, which may result from either natural infection or vaccination. In contrast, NSP-ELISA detects antibodies against non-structural proteins, which are produced only during active viral replication, thus indicating natural infection regardless of vaccination status.

Our results revealed that serotype O (66.1%) was the most prevalent (*p* < 0.05) serotype in large ruminants of the Pakistan–Afghanistan border region compared to other serotypes. The SP-ELISA further demonstrated that seroprevalence of serotype A (50%) was higher (*p* < 0.01) in the study area compared to Asia-1 (32%) serotype. Overall, 35.25% (282/800) samples of cattle and buffaloes were found positive for NSPs (Table 1).

### 3.2. Serotype-Specific Distribution

Our results revealed that serotype O (66.1%) was the most prevalent serotype, followed by serotype A (50%) and Asia-1 (32%). In cattle, serotype O was detected in 78% of samples, serotype A in 55%, and Asia-1 in 33%. In buffaloes, serotype O was detected in 55%, serotype A in 36%, and Asia-1 in 29% (Table 1).

### 3.3. Spatial Variations in Seroprevalence

**Figure 2:**
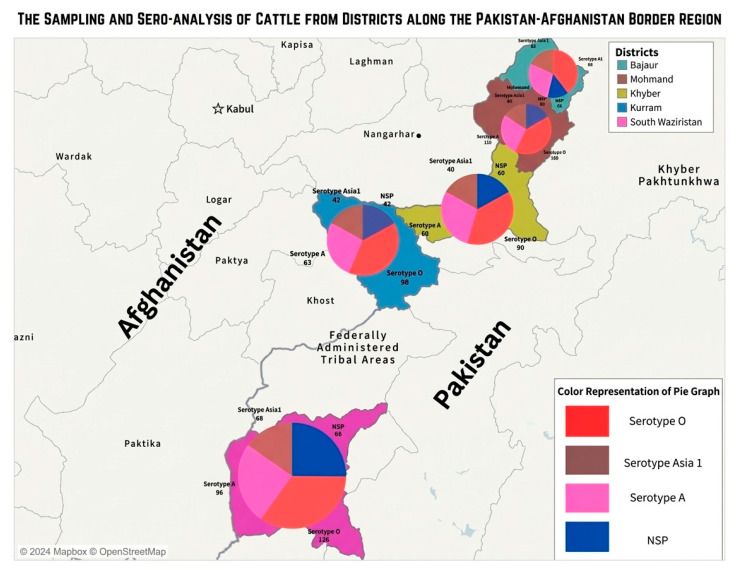
Serodiagnosis of FMDV antibodies to viral structural and nonstructural proteins in cattle (*n* = 610).

The findings of the current study revealed that significant (*p* < 0.05) variations in the seroprevalence of FMDV-SPs and -NSPs were recorded between various areas of Khyber Agency for both cattle and buffaloes. Similar patterns were recorded in Bajour, Mohmand, Kuram, and South Waziristan agencies. In cattle, the highest prevalence was observed in South Waziristan, followed by Kurram, Khyber, Mohmand, and Bajour (Figure 2). In buffaloes, the highest prevalence was observed in Khyber and Mohmand, followed by South Waziristan, Bajour, and Kurram (Figure 4).

### 3.4. Risk Factor Analysis

**Figure 3:**
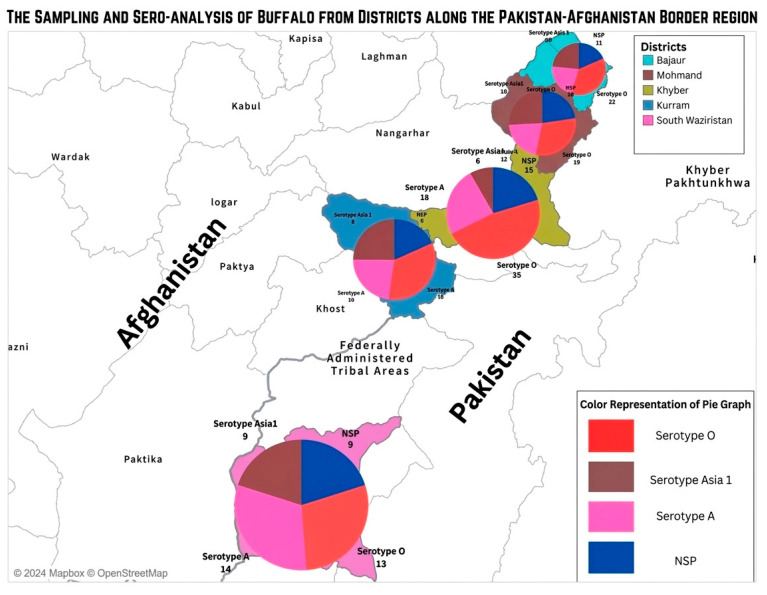
Serodiagnosis of FMD antibodies to viral structural and nonstructural proteins in buffaloes (*n* = 190).

The association of various risk factors with the prevalence of FMDV-NSP and -SPs in cattle and buffaloes is presented in Table 2 and Table 3 and Figure 2 and Figure 3, respectively.

Age-wise analysis indicated that, in cattle, the prevalence of antibodies against FMDV-NSP was significantly higher (*p* < 0.05) in the 1–2-year age group (40%) than in the 2–4-year (32%) and >4-year (33%) age groups (Table 2). In buffaloes, the results indicated that the prevalence of antibodies against non-structural protein was significantly (*p* < 0.05) lower in the >4 year age (19%) group as compared to the 1–2 year (35%) and 2–4 year age (31%) groups (Table 3). Likewise, FMDV-SPs were also more prevalent (*p* < 0.05) in young/adult animals compared to older animals.

Breed-wise analysis revealed that crossbreed and exotic cattle showed higher (*p* < 0.05) prevalence of FMDV-NSPs and -SPs as compared to local (pure) breed cattle. Contrary to this, crossbreed buffaloes were observed more (*p* < 0.05) prone to FMDV than local buffaloes.

Body condition assessment indicated that animals with poor body condition had significantly lower (*p* < 0.05) FMDV seroprevalence compared to those with good or intermediate body condition.

**Figure 4:**
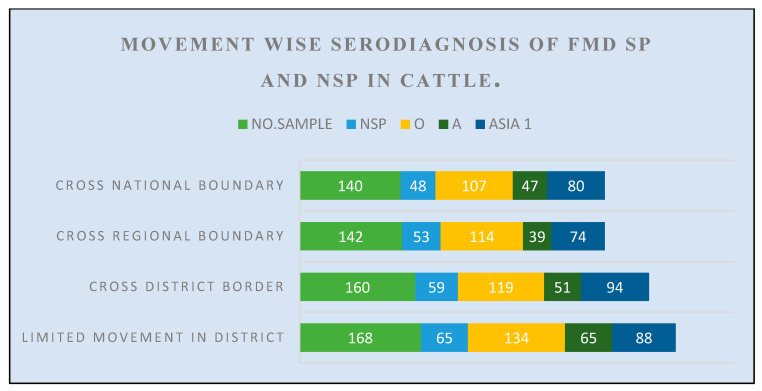
Movement-wise serodiagnosis of FMD SP and NSP in Cattle.

Other risk factors including season, sex, herd size, production system, and movement of cattle were also found significantly (*p* < 0.05) associated with the prevalence of FMDV-NSPs and -SPs in large ruminants of study area (Figure 4; Table 2 and Table 3).

## 4. Discussion

This study provides the first comprehensive sero-epidemiological assessment of FMDV in large ruminants along the Pakistan–Afghanistan border region. The overall NSP seroprevalence of 35.25% indicates that FMD is endemic in this region, with serotype O identified as the predominant circulating serotype (66.1%), followed by serotype A (50%) and Asia-1 (32%). These findings align with regional reports indicating serotype O as the most prevalent strain in Pakistan and neighboring countries [2]. The high prevalence of natural infection, combined with significant risk factors identified in this study, underscores the urgent need for targeted control interventions. We tested 800 serum samples from 610 cattle and 190 buffaloes for antibodies against structural and non-structural proteins of FMDV using SP- and NSP-ELISA. Our NSP results demonstrated a 35.25% apparent seroprevalence of FMDV in the study area, which is lower than the 67% reported by Ullah et al. [7] in the northern regions of Pakistan; this difference may be attributed to variations in species composition (the aforementioned study included small ruminants), sample size, and study area. Additionally, a recent survey by Zain et al. (2026) reported comprehensive PPRV seroprevalence data in small ruminants in district Quetta, Pakistan [18]. Some other reports from Pakistan reported 77.7% prevalence in buffaloes of Islamabad territory [19] and 13.84% in cattle of Bahawalpur district [20]. Afghanistan shares borders with Pakistan; east Afghanistan is very similar to Pakistan in terms of climatic conditions. A study conducted in Afghanistan reported 42% seropositive cattle in Puli Khumri, Khinjan, and Doshi districts of Baghlan province [1].

Although serotype O was the most prevalent, the substantial seroprevalence of serotype A (50%) and Asia-1 (32%) indicates that all three serotypes are actively circulating in the Pakistan–Afghanistan border region. This finding has critical implications for FMD control, as monovalent vaccines targeting only serotype O would be insufficient. Control programs should employ multivalent vaccines containing serotypes O, A, and Asia-1 to provide comprehensive protection against the circulating strains. This finding is consistent with Ullah et al. [21], who reported that serotype O is the dominant strain in Pakistan, followed by Asia-1 and A. The results of our study also agree with Chepkwony et al. [22], who found that FMDV type O was the most common in all areas the Pakistan–Afghanistan border region. The substantially higher SP seroprevalence (e.g., serotype O at 66.1%) compared to NSP seroprevalence (35.25%) reflects the distinct origins of these antibodies. SP antibodies may result from either vaccination or natural infection, whereas NSP antibodies specifically indicate natural infection. Although no coordinated mass vaccination campaign was in place at the time of sampling, sporadic vaccination by individual farmers—particularly in market-oriented herds—may have contributed to SP seropositivity. Additionally, in endemic settings such as the Pakistan–Afghanistan border region, animals may experience multiple FMDV infections with different serotypes over their lifetime. This repeated exposure can broaden SP antibody responses, while NSP antibody titers may decline between infections, falling below detectable levels [14].

Under the assumption of minimal vaccination in the study area, the high SP seroprevalence (66.1% for serotype O, 50% for serotype A, 32% for Asia-1) suggests that most animals are infected at least once in their lifetime with one or more of these serotypes. However, it is important to note that NSP antibodies indicate infection with any FMDV serotype, not only those included in our SP-ELISA. While serotypes O, A, and Asia-1 are the most prevalent in Pakistan and neighboring countries [2,21], the possibility of infection with other serotypes (e.g., SAT serotypes) cannot be entirely ruled out, though they are not typically reported in this region. The study found that cattle had a significantly higher (*p* < 0.05) prevalence of FMDV than buffaloes (37% vs. 30%), which is consistent with the study of Khan et al. [9], that reported 32.36% prevalence in cattle and 22.06% in buffalo in a district of Punjab Pakistan. The prevalence of FMDV varied among the different agencies and species of large ruminants. In cattle, the highest prevalence was observed in South Waziristan, followed by Kurram, Khyber, Mohmand, and Bajour. In buffaloes, the highest prevalence was observed in Khyber/Mohmand, followed by South Waziristan, Bajour, and Kurram. Some areas of Mohmand and South Waziristan were found free from FMDV in buffaloes. These differences may be related to the fact that South Waziristan and Mohmand do not have direct access to the neighboring Nangarhar province of Afghanistan. The observed variations in seroprevalence rates highlight geographical and environmental factors that may influence FMD Viral transmission dynamics [23]. González Gordon et al. reported that factors such as herd dynamics, cross-border movement, herd mobility, and contact at grazing/watering points contribute to the spread of FMDV.

Age was identified as a significant risk factor, with young animals (1–2 years) showing the highest NSP seroprevalence in both cattle (40%) and buffaloes (35%). This finding is consistent with previous studies [10,23] and can be explained by the waning of maternal antibodies, which typically decline between 6 and 12 months of age, leaving young animals susceptible before they develop active immunity through natural exposure or vaccination. Additionally, younger animals may have had fewer opportunities for prior exposure, making them more vulnerable to infection. The lower NSP seroprevalence observed in older animals (>4 years) compared to younger animals (1–2 years) may be explained by antibody decay over time. NSP antibodies have been shown to decline below detectable levels several years post-infection in the absence of re-exposure [14]. In contrast, a study by Kabir et al. [4] reported higher infection rates in older animals, which may reflect differences in vaccination status, exposure history, or management practices across study populations. The lower NSP seroprevalence in older animals may also be explained by antibody decay over time, as NSP antibodies have been shown to decline below detectable levels several years post-infection in the absence of re-exposure [14]. Additionally, the cross-sectional nature of our study captures a single time point and does not account for cohort effects, such as differences in FMDV exposure intensity across years or higher mortality in infected older animals.

We also showed that serotypes were least prevalent (*p* < 0.05) in large ruminants of the market-oriented production system, as compared to the smallholders subsistence and peri-urban commercial production systems in the study area. Studies have shown that large herd size is a risk factor for infectious diseases including FMDV [24]. As FMDV is a major transboundary disease, it is strongly associated with livestock movement and herd size; therefore, sedentary zones with direct market-oriented production systems are usually less affected and pose lower risks to other neighboring zones [4]. We further found that exotic and cross breed cattle had a higher prevalence of FMDV NSP- and -SP antibodies than local breed animals. This is consistent with other studies that have reported higher susceptibility of exotic and crossbred cattle to FMDV compared to indigenous breeds [25]. This difference may be explained by two factors. First, indigenous breeds may possess inherent genetic resistance to FMDV, developed through long-term natural selection in endemic environments [26,27]. Second, differences in management practices may influence exposure risk; exotic and crossbred animals are often reared in market-oriented systems with greater animal movement and contact, whereas local breeds are frequently kept in traditional systems with limited contact with outside herds [4]. While both factors likely contribute, the latter differences in exposure opportunity may play a particularly important role. It is well known that exotic animals (like Friesian cattle) are highly susceptible to tropical infections [26], whereas local breeds have a stronger immunity due to their adaptation to the climatic conditions in the region, which protects them from infectious diseases [27].

The finding that animals with poor body condition had lower FMDV seroprevalence is counterintuitive, as poor body condition is typically associated with compromised immunity and increased disease susceptibility. Two possible explanations may account for this observation. First, survival bias may have played a role: animals with poor body condition that became infected with FMDV may have experienced higher mortality prior to sampling, leading to their underrepresentation in our cross-sectional sample. This phenomenon, where severely affected animals are less likely to be captured in prevalence studies, is well documented in veterinary epidemiology [28]. Second, management practices may differ according to animal body condition. Farmers may preferentially trade or move healthier animals (in good body condition) to livestock markets, particularly during religious festivals such as Eid al-Adha, thereby increasing their exposure to FMDV. Conversely, animals in poor body condition are often confined to farms and have limited contact with outside herds, reducing their risk of exposure. Similar findings have been reported in other FMD risk factor studies, where animal movement and trade practices were identified as stronger predictors of seropositivity than body condition alone [7,23].

The high seroprevalence of FMDV antibodies in our current study suggests a substantial burden of FMDV in large ruminants in the Pakistan–Afghanistan border region. This underscores the urgent need for effective disease management strategies, such as implementing proper biosecurity measures, promoting the rearing of local animals, and enforcing movement restrictions.

Additionally, identifying the risk factors associated with FMDV transmission offers valuable insights into the local context. These insights are crucial for developing targeted interventions that can mitigate the spread of the disease and protect the livelihoods of local farmers and communities.

The study’s limitations include the cross-sectional design, which provides a snapshot of FMDV prevalence and risk factors but may not capture the temporal dynamics of the disease [23]. This design means that while we can see the prevalence at one specific point in time, we cannot observe how it changes or progresses throughout different seasons or years. Additionally, the sampling strategy employed a hybrid approach combining multistage cluster sampling with convenience elements due to security constraints and difficult terrain. While this allowed for feasible sample collection in a high-risk border region, it may limit the perfect representativeness of the sample for the entire animal population. The findings should therefore be interpreted with consideration of this practical limitation. Furthermore, NSP antibody titers may decline below detectable levels several years post-infection, particularly in the absence of re-exposure [14]. Therefore, the NSP seroprevalence of 35.25% reported in this study likely underestimates the true cumulative FMD viral infection rate in the study population. Samples that tested negative may represent either uninfected animals or animals with antibody levels below the assay’s detection threshold. Furthermore, it is important to acknowledge the uncertainties inherent in interpreting serological results in this setting. NSP seroprevalence likely underestimates the true cumulative infection rate due to antibody decay over time, as discussed above. Additionally, the absence of precise vaccination records for individual animals limits the ability to definitively distinguish between vaccine-induced and infection-induced SP antibody responses. While the reported seroprevalence values accurately reflect the proportion of samples reacting in each assay, these uncertainties should be considered when drawing conclusions about the precise burden of infection and the relative contributions of vaccination versus natural exposure. Future research could greatly benefit from longitudinal studies, which follow the same subjects over an extended period. This would allow us to monitor changes in Seroprevalence and risk factors over time, providing a deeper and more comprehensive understanding of the disease’s behavior and its triggers.

Additionally, further investigation into the genetic diversity of FMDV strains in the Pakistan–Afghanistan border region is crucial. Understanding the genetic variations among the strains can enhance our knowledge of viral circulation patterns. This insight is vital for developing targeted vaccines and control strategies that are specifically tailored to the prevalent strains in this high-risk area. Such research could significantly improve the effectiveness of disease management and prevention programs, ultimately safeguarding livestock health and supporting the livelihoods of local farmers.

## 5. Conclusions

In conclusion, this study reveals a high seroprevalence (35.25%) of FMDV in large ruminants along the Pakistan–Afghanistan border, with serotype O as the dominant strain. However, serotypes A and Asia-1 also show substantial circulation (50% and 32%, respectively), indicating that all three serotypes contribute significantly to the disease burden. Key risk factors include young age, crossbred/exotic breeds, large herd size, and animal movement associated with trade and religious festivals. These findings underscore the urgent need for government-led mass vaccination campaigns, targeted risk-based surveillance, and strict movement controls to mitigate disease spread and protect regional livestock economies. Implementation of such control measures, however, is likely to be complicated by ongoing armed conflict and security challenges in the border region, highlighting the need for context-specific strategies that can be effectively delivered even in unstable settings.

## Figures and Tables

**Table 1:** Overall seroprevalence of FMDV in the study area.

Species	N	NSP+ (%)	SP O+ (%)	SP Asia1+ (%)	SP A+ (%)
Cattle	610	(225) 37	(474) 78	(202) 33	(336) 55
Buffaloes	190	(57) 30	(104) 55	(56) 29	(68) 36
Total	800	(282) 35	(578) 72	(258) 32	(404) 50

**Table 2:** Serodiagnosis of FMD antibodies to viral structural and non-structural proteins in cattle (*n* = 610).

Variables	Category	No of Samples	Seroprevalence of FMD Proteins				
			NSP No.% (95%CI)	Serotype O No./% (95%CI)	Serotype A No./% (95%CI)	Serotype Asia1 No./% (95%CI)	*p*-Value
Age	1–2 Year	321	130/40 (35.1–45.2)	254/79 (74.5–83.1)	93/29 (24.2–34.2)	160/50 (44.5–55.5)	0.042 *
	2–4 Year	154	50/32 (25.3–40.1)	122/79 (72.1–85.3)	77/50 (42.1–58.0)	97/63 (55.2–70.5)	
	>4 Year	135	45/33 (25.8–41.5)	98/72 (64.2–79.8)	32/24 (17.2–31.8)	79/58 (49.8–66.2)	
Sex	Male	258	75/29 (23.8–34.9)	194/75 (69.4–80.3)	104/40 (34.5–46.2)	180/70 (64.2–75.5)	0.001 *
	Female	352	149/42 (37.2–47.5)	280/79 (75.0–83.2)	98/28 (23.5–33.1)	156/44 (39.2–49.5)	
Breed	Crossbred	167	77/46 (38.7–54.0)	135/81 (74.2–86.5)	79/47 (39.7–55.1)	91/54 (46.8–62.0)	0.001 *
	Exotic	58	41/71 (57.8–81.5)	53/91 (81.2–97.2)	27/46 (33.8–60.2)	37/64 (50.5–76.2)	
	Local	385	107/28 (23.5–32.5)	286/74 (69.8–78.5)	96/25 (20.9–29.8)	208/54 (49.1–59.0)	
Season	Spring	166	46/28 (21.5–35.2)	139/84 (77.5–89.2)	53/32 (25.2–39.5)	80/48 (40.5–55.8)	0.001 *
	Summer	144	67/46 (38.2–55.1)	126/87 (81.2–92.8)	59/41 (33.2–49.5)	102/71 (63.2–78.5)	
	Autumn	154	71/46 (38.2–54.5)	107/69 (61.5–76.8)	94/53 (45.2–61.2)	85/55 (47.2–63.2)	
	Winter	146	41/28 (21.2–35.8)	102/70 (62.2–77.5)	41/28 (21.2–35.8)	69/47 (39.2–55.5)	
Production system	Smallholders Subsistence	426	165/39 (34.2–43.5)	365/86 (82.2–89.2)	151/35 (30.8–40.2)	255/60 (55.2–64.8)	0.001 *
	Market-oriented	174	56/33 (26.2–40.5)	102/59 (51.5–66.2)	48/27 (21.2–34.5)	76/44 (36.8–51.5)	
	Peri-urban Commercial	10	4/40 (12.2–73.8)	7/70 (35.2–93.5)	3/30 (6.5–65.2)	5/50 (19.2–80.8)	
Herd size	Small	389	132/34 (29.2–38.8)	309/79 (75.2–83.2)	134/34 (29.8–39.2)	249/64 (59.2–68.8)	0.008 *
	Medium	181	69/38 (31.2–45.5)	151/83 (77.2–88.5)	56/31 (24.2–38.5)	71/39 (32.2–46.5)	
	Large	40	24/60 (43.5–75.2)	14/35 (21.2–52.5)	12/30 (16.5–46.2)	16/40 (25.2–57.5)	
Body condition	Poor	198	59/30 (23.8–36.8)	159/80 (74.2–85.8)	59/30 (23.8–36.8)	86/43 (36.5–50.5)	0.021 *
	Intermediate	191	69/36 (29.5–43.2)	121/63 (56.2–70.2)	61/32 (25.5–39.2)	111/58 (51.2–65.2)	
	Good	221	97/44 (37.5–50.8)	194/88 (83.2–92.2)	82/37 (31.2–43.8)	139/63 (56.5–69.5)	

* *p*-values derived from Chi-square test; values <0.05 were considered statistically significant. All 95% confidence intervals were calculated using the Wilson score method.

**Table 3:** Serodiagnosis of FMD antibodies to viral structural and non-structural proteins in buffaloes (*n* = 190).

Variables	Category	No of Samples	Seroprevalence of FMD Proteins				
			NSP No.%(95%CI)	Serotype O No./% (95%CI)	Serotype A No./% (95%CI)	Serotype Asia1 No./% (95%CI)	*p*-Value
Age	1–2 Year	95	33/35 (25.5–45.2)	61/64 (54.2–73.5)	32/34 (24.5–44.2)	37/39 (29.5–49.2)	0.035 *
Sex	2–4 Year	49	15/31 (18.5–45.2)	30/61 (46.5–75.2)	15/31 (18.5–45.2)	19/39 (25.2–54.5)	
	>4 Year	46	9/19 (9.5–34.2)	13/28 (16.2–43.5)	9/19 (9.5–34.2)	12/26 (14.2–41.5)	
	Male	72	25/35 (24.2–47.5)	39/54 (42.2–66.2)	23/32 (21.2–44.5)	38/53 (41.2–65.2)	0.224
Breed	Female	118	32/27 (19.2–36.5)	65/55 (46.2–64.5)	33/28 (20.5–37.2)	68/58 (48.5–67.5)	
	Local	41	13/32 (18.5–48.5)	30/73 (57.2–86.2)	16/39 (24.5–55.2)	14/34 (20.2–50.5)	0.018 *
	Cross breed	149	44/29 (22.2–37.2)	74/50 (41.2–58.5)	40/27 (19.5–35.2)	54/36 (28.2–44.5)	
	Exotic	35	10/28 (15.2–46.5)	25/71 (54.2–85.5)	14/40 (24.2–58.2)	19/54 (37.2–71.2)	
Season (months)	Spring	35	10/28 (15.2–46.5)	25/71 (54.2–85.5)	14/40 (24.2–58.2)	19/54 (37.2–71.2)	0.042 *
	Summer	64	23/36 (24.2–49.5)	43/67 (54.2–78.5)	20/31 (20.2–44.2)	22/34 (23.2–47.5)	
	Autumn	49	15/31 (18.5–45.2)	20/41 (27.2–56.5)	12/24 (13.2–39.5)	14/29 (16.5–43.5)	
	Winter	42	9/21 (10.5–37.5)	16/38 (28.2–59.2)	10/24 (12.5–39.2)	13/31 (18.2–48.5)	
Production system	Smallholders Subsistence	134	43/32 (24.2–41.2)	81/60 (52.2–69.5)	46/34 (26.2–43.2)	52/39 (30.5–48.2)	0.003 *
	Market-oriented Production	56	14/25 (14.5–38.5)	23/41 (28.5–55.2)	10/18 (8.5–30.5)	16/28 (17.2–42.5)	
Body condition	Poor	49	12/24 (13.5–39.5)	22/45 (31.2–60.2)	11/22 (12.5–37.5)	10/20 (10.5–34.5)	0.047 *
	Intermediate	54	15/28 (16.5–42.5)	29/54 (40.5–67.5)	17/31 (19.5–45.5)	25/46 (33.2–60.2)	
	Good	87	31/36 (26.5–47.2)	53/61 (50.5–71.2)	28/32 (22.5–43.5)	33/38 (28.5–49.5)	

* *p*-values derived from Chi-square test; values <0.05 were considered statistically significant. All 95% confidence intervals were calculated using the Wilson score method.

## Data Availability

The original contributions presented in this study are included in the article. Further inquiries can be directed to the corresponding authors.
